# Pyogenic Liver Abscess Risk in Patients With Newly Diagnosed Type 2 Diabetes Mellitus: A Nationwide, Population-Based Cohort Study

**DOI:** 10.3389/fmed.2021.675345

**Published:** 2021-05-12

**Authors:** Tzu-Yuan Wang, Hsueh-Chou Lai, Hsin-Hung Chen, Mei-Lin Wang, Ming-Chia Hsieh, Chwen-Tzuei Chang, Rong-Hsing Chen, Chun-Wei Ho, Yi-Chin Hung, Juei-Yu Tseng, Cheng-Li Lin, Chia-Hung Kao

**Affiliations:** ^1^Intelligent Diabetes Metabolism and Exercise Center, Department of Internal Medicine, China Medical University Hospital, Taichung, Taiwan; ^2^School of Medicine, China Medical University, Taichung, Taiwan; ^3^Division of Hepato-Gastroenterology, Department of Internal Medicine, China Medical University Hospital, Taichung, Taiwan; ^4^Division of Endocrinology and Metabolism, Department of Internal Medicine, Asia University Hospital, Taichung, Taiwan; ^5^School of Medicine, Chung Shan Medical University, Taichung, Taiwan; ^6^Chung Sheng Clinic, Nantou, Taiwan; ^7^Department of Nursing, Hung Kuang University, Taichung, Taiwan; ^8^Management Office for Health Data, China Medical University Hospital, Taichung, Taiwan; ^9^College of Medicine, China Medical University, Taichung, Taiwan; ^10^Graduate Institute of Biomedical Sciences Science, School of Medicine, College of Medicine, China Medical University, Taichung, Taiwan; ^11^Department of Nuclear Medicine and Positron Emission Tomography (PET) Center, China Medical University Hospital, Taichung, Taiwan; ^12^Department of Bioinformatics and Medical Engineering, Asia University, Taichung, Taiwan; ^13^Center of Augmented Intelligence in Healthcare, China Medical University Hospital, Taichung, Taiwan

**Keywords:** pyogenic liver abscess, newly diagnosed type 2 diabetic mellitus, Klebsiella pneumonia, acarbose, National Health Insurance Research Database

## Abstract

**Background:** To date, no comprehensive epidemiological study exists on pyogenic liver abscess (PLA) risk in patients with newly diagnosed type 2 diabetes mellitus (T2DM) worldwide.

**Methods:** We conducted a retrospective cohort study by using data from Taiwan National Health Insurance Research Database (NHIRD) to examine the association between newly diagnosed T2DM and PLA. The T2DM cohort included patients newly diagnosed as having T2DM (ICD-9-CM:250) from 2000 to 2009, with follow-up until December 31, 2011. The comparison cohort was then recruited through 1:4 random frequency matching with the T2DM cohort. Finally, the adjusted hazard ratios for PLA were compared between the T2DM and comparison cohorts, which included 44,728 patients with T2DM and 178,912 patients without DM respectively.

**Results:** In T2DM cohort, 166 patients were diagnosed as having PLA (incidence rate = 5.87 per 10,000 person-years) and in comparison cohort, 238 patients were diagnosed as having PLA (incidence rate = 2.06 per 10,000 person-years). The T2DM cohort exhibited higher PLA risk than did the comparison cohort (hazard ratio = 2.83, 95% confidence interval = 2.32–3.46). Furthermore, the adjusted hazard ratio for PLA risk in T2DM cohort was the highest in those who were younger, man and with duration of DM <2 years. In the T2DM cohort, the most common PLA causative agent was Klebsiella pneumonia (KP). In addition, PLA risk was high in T2DM patients with gallstone and cholecystitis. Compared with comparison cohort, patients with T2DM prescribed acarbose has a lower PLA risk, however glyburide significantly increased PLA risk in T2DM cohort.

**Conclusion:** In patients with newly diagnosed T2DM, PLA risk was high and acarbose might reduce PLA risk.

## What Is Already Known About This Topic?

To date, no comprehensive epidemiological study exists on pyogenic liver abscess (PLA) risk in patients with newly diagnosed type 2 diabetes mellitus (T2DM) worldwide.

## What Does This Article Add?

In patients with newly diagnosed T2DM, PLA risk was high and acarbose might reduce PLA risk.

## Introduction

Pyogenic liver abscess (PLA) is a serious infectious and life-threatening disease with a low but gradually increasing annual incidence rate. The incidence rate was only 1.0 per 100,000 person-years from 1997 to 2002 in Denmark (1) and 2.3 per 100,000 person-years from 1999 to 2003 in Canada (2). However, PLA incidence rate is much higher in Taiwan. A nationwide analysis from the National Health Insurance Research Database (NHIRD) data from 2000 through 2011 demonstrated that the annual incidence of PLA for all ages groups increased gradually from 10.83 per 100,000 person-years in 2000 to 15.45 per 100,000 person-years in 2011 (3). Moreover, during 2000–2011, the average annual incidence was 13.52 per 100,000 person-years. PLA has become a health problem in the Taiwanese society.

PLAs often results from the complications of biliary tract infection, and globally, the most common PLA pathogen is *Escherichia coli* (*E. coli*) (4). However, accumulating evidence in Taiwan has shown a direct relationship between PLA and Klebsiella pneumoniae (KP) in patients with Diabetes Mellitus (DM) without biliary tract infection (5–7). Over 1981–1993, the medical records of 146 adults treated for PLA in the hospitals in Taipei were retrospectively investigated. Of all PLA cases, 78% were due to KP, and more patients with PLA causing by KP were observed to exhibit DM than did patients with non- KP PLA (66 vs. 19%) (5). In another study, 182 patients with PLAs treated at Kaohsiung Veterans General Hospital, Taiwan from 1990 to 1996 were enrolled. Of these patients, 87.9% had PLAs caused by KP. In addition, patients with PLA causes by KP exhibited higher DM and glucose intolerance incidence rate than did patients with non-KP PLA (75 vs. 4.5%) (6). Furthermore, a study in Taiwan demonstrated that of 1,522 adults with PLA, 35.3% had DM, 10.8% had complications, 15.4% received mechanical ventilation and 23.7% were provided intensive care (3). These data suggest that PLA caused by KP is closely associated with DM. Furthermore, several aspects are believed to be affected in patients with DM such as polymorphonuclear leukocyte function inhibition, leukocyte adherence, chemotaxis, phagocytosis and impairment of antioxidant systems involved in bactericidal activity (7).

To date, no comprehensive epidemiological study exists for PLA risk in patients with newly diagnosed T2DM worldwide. Thus, we conducted a large retrospective cohort study by using the health care service file from the NHIRD to examine the association between newly diagnosed T2DM and PLA risk. We compared the clinical manifestations, clinical outcomes and drug treatments recorded for patients with PLA in the DM and non-DM groups.

## Patients and Methods

### Data Sources

We used the Longitudinal Health Insurance Database (LHID) for this study. The LHID is a subset of NHIRD which contains all claims data from the Taiwan National Health Insurance (NHI) beneficiaries (including nearly 23 million Taiwan residents). The NHI system of Taiwan was established in 1995 and covers more than 99.6% of the Taiwanese population; this system's claims data are released as the NHIRD (8). The LHID was contains 1996–2000 data of random 1 million insured people randomly selected from NHI beneficiaries. According to the Taiwan government report, the age and sex distribution are not different for the LHID and NHIRD. The LHID features claims data including a registry for beneficiaries, records of clinic care and hospital care, drug prescriptions and other medical services and these data are renewed annually. The disease record system of clinic and hospital care is based on International Classification of Diseases, Ninth Revision, Clinical Modification (ICD-9-CM). Before the data are releasing for research, the Taiwan government implements privacy protection for insured individuals, removes the original identification numbers and provides an encoded serial numbers for insured people to link their claims data. Furthermore, this study was approved by the Ethics Review Board of China Medical University (CMUH104-REC2-115-AR4).

### Study Population

**Figure 1:**
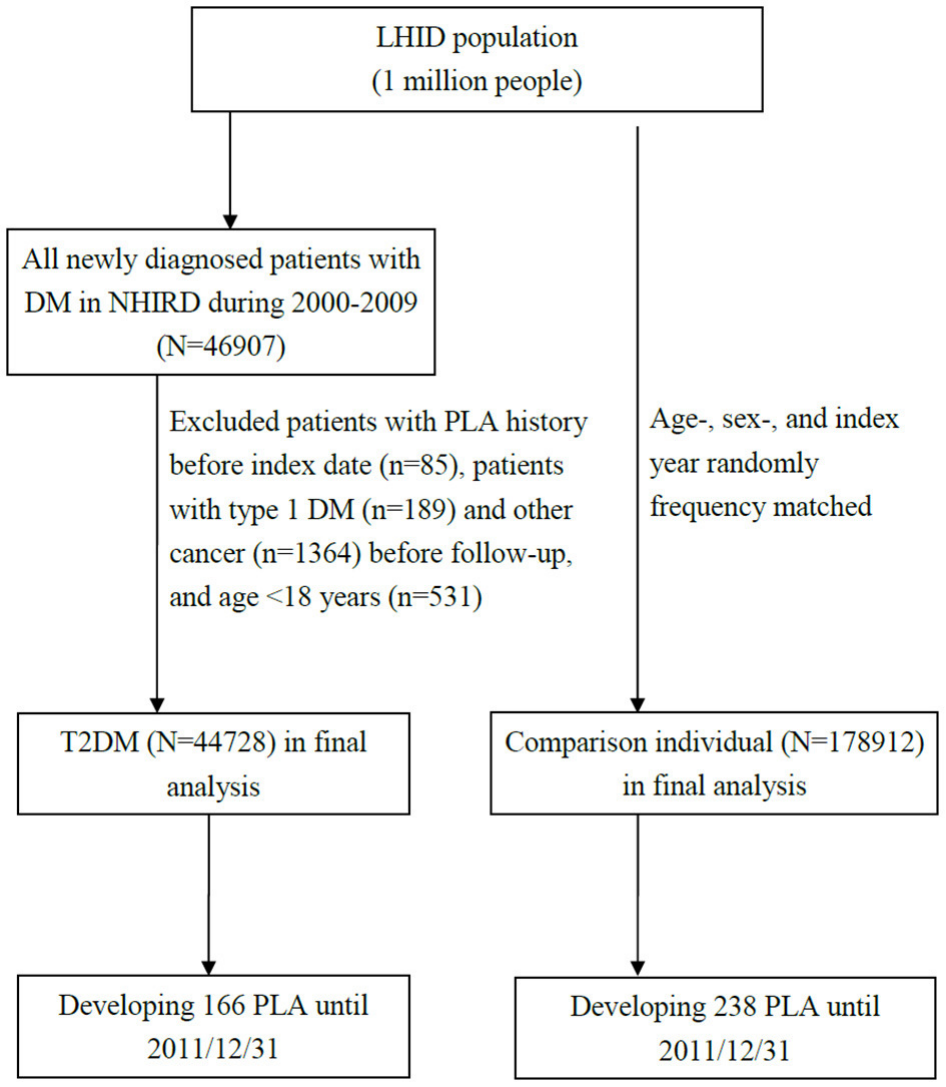
Flow of cohort selection.

We extracted a T2DM and comparison cohorts from the aforementioned databases and compared PLA risks between them. In the T2DM cohort, patients newly diagnosed as having T2DM (ICD-9-CM 250) from 2000 to 2009 at two outpatient records within 1 year were enrolled from the LHID. We used date of initial T2DM diagnosis as the index date. In the comparison, individuals without a history of DM from LHID were randomly 1:4 frequency-matched with the T2DM cohort, the matching was based on the age of cohort entry (per 5 years) and sex. The index date for the patients in the comparison cohort was created by matching the year of the index date of patients in the T2DM cohort. We excluded participants age <18 years or type 1 DM, PLA or cancer before the index date. The study outcome of the interest was the occurrence of PLAs (ICD-9-CM 572.0) at inpatient record. For both cohorts follow-up commenced at index date and was terminated when the individual withdrew insurance, when PLA occurred or on December 31, 2011. Figure 1 depicts the flow for cohort selection.

### Criteria and Definitions

Age, sex and PLA-associated comorbidities were considered confounding factors in this study. The PLA-associated comorbidity was defined as individuals with a comorbidity diagnosis history before the index date and comorbidities included gallstone (ICD-9-CM 574), cholecystitis (ICD-9-CM 575.0 and 575.10) and cholangitis (ICD-9-CM 576.1).

This study also investigated the effect of DM medication on PLA risk in patients with T2DM. Patients administered DM medications during the observation time were grouped according to the type of medication used. The DM medications included metformin, glyburide, glimepiride, gliclazide, glipizide, thiazolidinedione (TZD; including rosiglitazone and pioglitazone), dipeptidyl peptidase-4 inhibitor (DPP4i) and acarbose.

The microorganisms were identified on the same date as the first diagnosis of PLA during hospitalization. The microorganisms included *Staphylococcus* (ICD-9-CM 038.0 and 041.1), *E.coli* (ICD-9-CM 038.42 and 041.4), *Streptococcus* (ICD-9-CM 038.0 and 041.0), *Pneumococcus* (ICD-9-CM 038.1 and 041.2), *KP* (ICD-9-CM 041.3), *Proteus* (ICD-9-CM 041.6), gram-negative bacteria (ICD-9-CM 038.40, 038.49, and 041.85), and other/unspecified bacteria (ICD-9-CM 038.8, 038.9, and 041).

### Statistical Analysis

The study population characteristics are presented as the means and standard deviation (SDs) for age and as numbers and percentages for sex, comorbidity and DM. In each group, PLA incidence rate was calculated as number of patients divided by the sum of follow-up duration (per 10,000 person-years). The PLA cumulative incidence curve was measured for the T2DM cohort and comparison cohorts by using Kaplan-Meier method. We assessed the difference between the curves for the two study cohorts by using log-rank test. PLA risk in the T2DM and comparison cohorts are presented as hazard ratios (HRs) and 95% confidence intervals (CIs) which were estimated by using a Cox proportional hazard regression model. We also analyzed the PLA risk in individuals with and without DM stratified by age, sex, comorbidities and follow-up duration. All analyses were performed on SAS (version 9.4; SAS Institute, Cary, NC, USA) and the cumulative curve was plotted using R (R Foundation for Statistical Computing, Vienna, Austria). To assess the distribution difference for the individuals with and without DM, we used the two sample t- test for age and the chi-square test for sex and comorbidity. We calculated the incidence density of PLA for each study cohort, with statistical significance defined as a two-tailed *p* of < 0.05.

## Results

We enrolled a cohort of 44,728 patients with T2DM and 178,912 age and sex matched individuals (Table 1). Except for cholangitis, the rates of gallstone and cholecystitis occurrences in patients with T2DM were significantly higher than the proportions in comparison patients (p < 0.05). In patients with T2DM, the most used medications were metformin (60.4%), glimepiride (36.5%) and gliclazide (31.2%).

**Table 1:** Baseline demographic status and comorbidity for comparison and T2DM cohorts.

**Variable**	**Comparison cohort** ***N* = 178,912 (%)**	**T2DM cohort** ***N* = 44,728 (%)**	***p*-value**
Age, years (SD)^*^	55.5 (14.1)	55.6 (13.9)	0.1204
<40	23,404 (13.1)	5,851 (13.1)	
40–59	90,192 (50.4)	22,548 (50.4)	
60–79	57,776 (32.3)	14,444 (32.3)	
≧80	7,540 (4.2)	1,885 (4.2)	
**Sex**			>0.99
Female	82,008 (45.8)	20,502 (45.8)	
Male	96,904 (54.2)	24,226 (54.2)	
**Comorbidity**			
Gallstone	5,625 (3.1)	1,684 (3.8)	<0.0001
Cholecystitis	797 (0.4)	261 (0.6)	0.0001
Cholangitis	585 (0.3)	163 (0.4)	0.2198
**Anti DM medication**			
Metformin		27,030 (60.4)	
Glyburide		10,595 (23.7)	
Glimepiride		16,338 (36.5)	
Gliclazide		13,939 (31.2)	
Glipizide		7,071 (15.8)	
TZD		7,354 (16.4)	
DPP4i		4,545 (10.2)	
Acarbose		7,578 (16.9)	
Others		6,177 (13.8)	

* *t-test*.

*TZD, thiazolidinedione; DPP4i, Dipeptidyl peptidase-4 inhibitor*.

**Figure 2:**
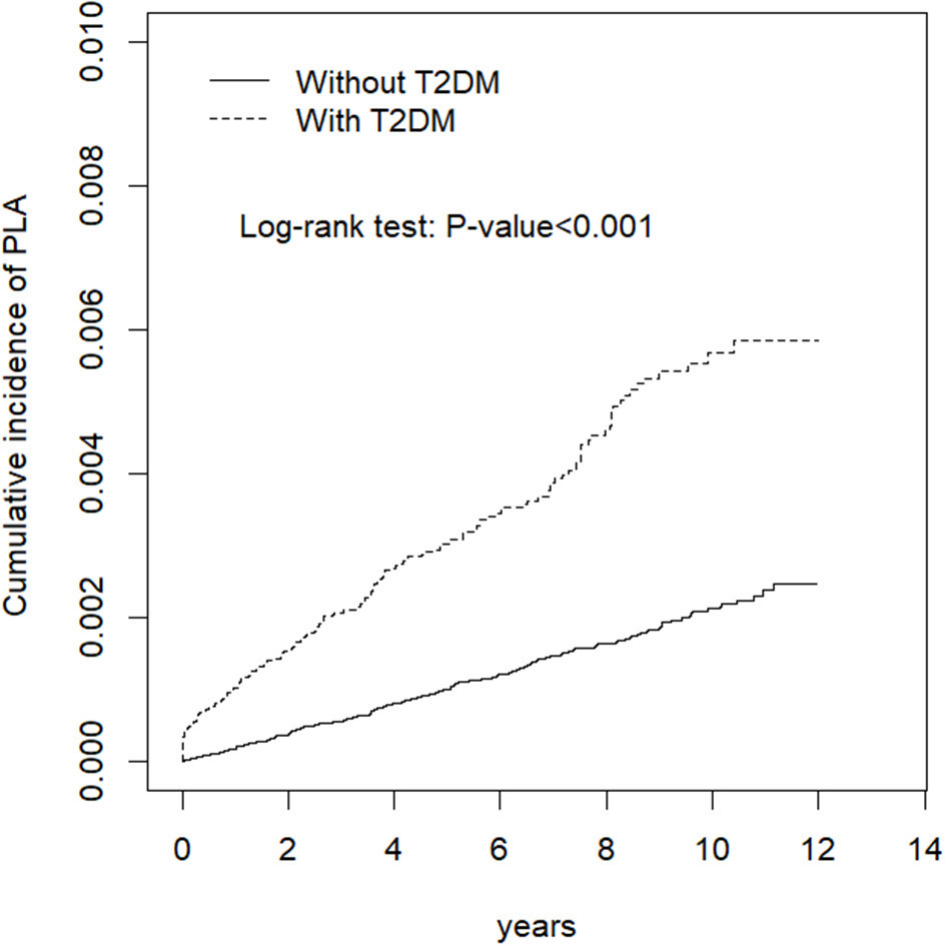
Cumulative incidence of PLA in the T2DM and comparison cohort.

PLA incidence rate was 2.06 and 5.87 per 10,000 person-years in the comparison and T2DM cohorts, respectively (Table 2). Figure 2 also shows cumulative incidence was significantly higher in the T2DM cohort than in the comparison cohort (P <0.0001, log-rank test). After adjustments for age, sex, gallstone occurrences, cholecystitis occurrences and cholangitis occurrences, the T2DM cohort has a higher PLA risk than did the comparison cohort (HR = 2.83, 95% CI = 2.32–3.46). Moreover, PLA risk was higher in older patients, man (HR = 1.69, 95% CI = 1.38–2.07), patient with gallstone (HR = 2.63, 95% CI = 1.81–3.80) and patients with cholangitis (HR = 4.62, 95% CI = 2.44–8.74).

**Table 2:** HRs and adjusted HRs for PLA in the study cohort.

**Variable**	**Event**	**PYs**	**Rate**	**HR (95% CI)**	**aHR^†^ (95% CI)**
**DM**					
No	238	1,152,686	2.06	Ref	Ref
Yes	166	282,924	5.87	2.84 (2.33–3.46)	2.83 (2.32–3.46)
**Age group**					
<40	23	197,460	1.16	Ref	Ref
40–59	174	738,840	2.36	2.02 (1.31–3.12)	1.99 (1.29–3.07)
60–79	183	458,783	3.99	3.42 (2.22–5.28)	3.29 (2.13–5.09)
≧80	24	40,528	5.92	5.01 (2.83–8.89)	4.55 (2.55–8.10)
**Sex**					
Female	145	675,211	2.15	Ref	Ref
Male	259	760,399	3.41	1.58 (1.29–1.94)	1.69 (1.38–2.07)
**Gallstone**					
No	362	1,394,425	2.60	Ref	Ref
Yes	42	41,185	10.2	3.91 (2.84–5.39)	2.63 (1.81–3.80)
**Cholecystitis**					
No	400	1,429,802	2.80	Ref	Ref
Yes	4	5,808	6.89	2.45 (0.91–6.55)	0.77 (0.28–2.14)
**Cholangitis**					
No	391	1,431,849	2.73	Ref	Ref
Yes	13	3,761	34.56	12.54 (7.21–21.79)	4.62 (2.44–8.74)

† *Model adjusted for age, sex, gallstone, cholecystitis, and cholangitis*.

*PYs, person-years; Rate, incidence rate, per 10,000 person-years*.

In patients with T2DM, Table 3 presents the PLA risk associated with various DM medications. After adjustments for sex, age, comorbidity, and DM medication uses, patients with DM administered glyburide were found to exhibit a significantly increased PLA risk compared with patients not administered glyburide (HR = 1.84, 95% CI = 1.27–2.66). Compared with those not administered acarbose, administered acarbose had lower PLA risk (HR = 0.51, 95% CI = 0.29–0.90).

**Table 3:** HRs and adjusted HRs for PLA in the T2DM cohort according to DM medications.

**Variable**	**Event**	**PYs**	**Rate**	**HR (95% CI)**	**aHR^†^ (95% CI)**
**Metformin**					
No	81	107,634	7.53	Ref	Ref
Yes	85	175,290	4.85	0.65 (0.48–0.88)	0.69 (0.47–1.01)
**Glyburide**					
No	111	204,240	5.43	Ref	Ref
Yes	55	78,684	6.99	1.33 (0.96–1.85)	1.84 (1.27–2.66)
**Glimepiride**					
No	118	171,670	6.87	Ref	Ref
Yes	48	111,254	4.31	0.64 (0.46–0.89)	0.80 (0.54–1.18)
**Gliclazide**					
No	109	183,498	5.94	Ref	Ref
Yes	57	99,426	5.73	0.99 (0.72–1.37)	1.37 (0.94–2.00)
**Glipizide**					
No	141	230,673	6.11	Ref	Ref
Yes	25	52,251	4.78	0.81 (0.53–1.23)	0.89 (0.57–1.41)
**TZD**					
No	146	226,616	6.44	Ref	Ref
Yes	20	56,308	3.55	0.57 (0.36–0.91)	0.87 (0.52–1.45)
**DPP4i**					
No	166	249,575	6.65	Ref	Ref
Yes	0	33,349	0	–	–
**Acarbose**					
No	151	227,523	6.64	Ref	Ref
Yes	15	55,401	2.71	0.42 (0.25–0.71)	0.51 (0.29–0.90)
**Others**					
No	143	239,250	5.98	Ref	Ref
Yes	23	43,674	5.27	0.90 (0.58–1.39)	1.17 (0.73–1.89)

† *Model adjusted for age, sex, gallstone, cholecystitis, cholangitis and each DM medications.*

*PYs, person-years; Rate, incidence rate, per 10,000 person-years*.

*TZD, thiazolidinedione; DPP4i, Dipeptidyl peptidase-4 inhibitor*.

Table 4 presents the results of our demographic factor and comorbidity-stratified analysis for PLA risk in our cohort. Except for those ≥80 years, all patients with DM exhibited an increased HR for PLA risk being particularly high in those aged <40 years (HR = 7.55, 95% CI = 3.20–17.8). Compared with the comparison cohort. T2DM was significantly associated with a higher PLA risk in both women (HR = 2.43, 95% CI = 1.73–3.40) and men (HR = 3.10, 95% CI = 2.43–3.97). Moreover, PLA risk was higher in patients with T2DM without comorbidities than in the comparison cohort without comorbidities.

**Table 4:** Demographic factor and comorbidity-stratified analysis of HR for PLA risk in comparison and DM cohort.

	**Comparison group**			**T2DM group**			**Adjusted HR** **(95% CI)**
**Variables**	**Event**	**PYs**	**Rate**	**Event**	**PYs**	**Rate**	
**Age group**							
<40	8	157,747	0.51	15	39,713	3.78	7.55 (3.20–17.8)
40–59	93	592,371	1.57	81	146,469	5.53	3.47 (2.57–4.67)
60–79	120	369,438	3.25	63	89,345	7.05	2.16 (1.59–2.93)
≧80	17	33,130	5.13	7	7,398	9.46	1.80 (0.74–4.33)
**Sex**							
Female	91	541,816	1.68	54	133,395	4.05	2.43 (1.73–3.40)
Male	147	610,870	2.41	112	149,529	7.49	3.10 (2.43–3.97)
**Gallstone**							
No	209	1,120,990	1.86	153	273,435	5.60	3.02 (2.45–3.72)
Yes	29	31,696	9.15	13	9,489	13.7	1.58 (0.82–3.05)
**Cholecystitis**							
No	235	1,148,290	2.05	165	281,512	5.86	2.86 (2.34–3.49)
Yes	3	4,396	6.82	1	1,412	7.08	1.03 (0.11–10.15)
**Cholangitis**							
No	227	1,149,734	1.97	164	282,115	5.81	2.95 (2.41–3.60)
Yes	11	2,953	37.26	2	809	24.73	0.70 (0.15–3.21)

*Model adjusted for age, sex, gallstone, cholecystitis, and cholangitis*.

*Rate, incidence rate, per 10,000 person-years*.

In Table 5, the PLA risk is compared between the T2DM and comparison cohorts for various follow-up durations. Compared with the individual without T2DM, PLA risk was the highest <2 years after DM diagnosis (HR = 4.02, 95% CI = 2.87–5.63). PLA risk was nearly 2-fold higher 2–4 years (HR = 2.74, 95% CI = 1.85–4.06) and ≧4 years (HR = 2.13, 95% CI = 1.55–2.95) after DM diagnosis.

**Table 5:** HRs for PLA, according to follow-up duration.

	**Comparison cohort**			**T2DM cohort**			**Adjusted HR (95% CI)**
**Follow-up duration**	**Event**	**PY**	**Rate**	**Event**	**PY**	**Rate**	
Year <2	68	352,319	1.93	68	87,337	7.79	4.02 (2.87–5.63)
Year 2–4	62	301,789	2.05	42	74,365	5.65	2.74 (1.85–4.06)
Year ≧ 4	108	498,578	2.17	56	121,223	4.62	2.13 (1.55–2.95)

*Model adjusted for age, sex, gallstone, cholecystitis, and cholangitis*.

*PYs, person-years; Rate, incidence rate, per 10,000 person-years*.

Of all PLA patients with PLAs, 80.7% with T2DM and 84.9% without T2DM demonstrated positive microorganism test results (Table 6). KP was prevalent microorganisms in both the T2DM (30.7%) and comparison (27.8%) cohorts.

**Table 6:** Microorganism test results of patients with PLA (*N* = 404).

	**Microorganism test**					
	**Positive**, ***n*** **(%)**				**Negative, *n* (%)**	**Total, *n***
	**All**	**KP**	***E. coli***	**Others**		
**DM**						
With DM	92 (80.7)	35 (30.7)	10 (8.7)	47 (41.2)	22 (19.3)	114
Without	107 (84.9)	35 (27.8)	22 (17.5)	50 (39.7)	19 (15.1)	126

## Discussion

Our report is the first retrospective population-based cohort study that use the NHIRD in Taiwan to investigate the relationship between newly diagnosed T2DM and subsequent PLA risk. The analyzed data confirmed that patients with newly diagnosed T2DM had a higher PLA risk than did those without T2DM. Here, the T2DM cohort included patients newly diagnosed as having T2DM from 2000 to 2009, who were then followed until 2011. The PLA incidence rate reached 5.87 and 2.06 per 10,000 person-years in the T2DM and comparison cohorts, respectively. Patients with newly diagnosed T2DM had a 2.83-fold higher PLA risk than did patients with PLA but without T2DM. Increased PLA risk in patients with DM was also reported in Danish large nationwide case-control study (9), according to which individuals with DM had a 3.6-fold increased PLA risk. Moreover, in Canada, an epidemiological study showed that the relative risk of PLA to be 11.1 in patients with DM (2). The potential reasons underlying this observation are as follows: First, patients with diabetes have a higher risk of susceptibility to various common or serious infections (10, 11).

Although the pathophysiology of infections related to DM remains unclear, they may be caused by the hyperglycemic environment which results in immune dysfunction. Defects in leukocyte function, such as adherence, chemotaxis and phagocytosis have been reported in patients with DM (12). Second, complement deficiencies such as C3 or C4 deficiencies which are potentially associated with decreased cytokine response and reduced immobilization of polymorphonuclear leukocyte have been observed in many patients with DM (13), and the antioxidant systems related to antibacterial activity may also be damaged. KP is a major causative agent of PLA in Taiwan, particularly in patients with DM (14, 15).

A Taiwanese study on the impact of glycemic control on the characteristics of PLA caused by KP in patients with DM reported that patients with uncontrolled glycemia tended to be younger, and have higher PLA rates of cryptogenic invasive PLA, caused by KP and metastatic infection than did those with good glycemic control (7). In our study, we found that the adjusted HR for PLA risk in the DM cohort was the highest in the youngest age group compared with the comparison cohort, and that men had a greater adjusted HR than women did, as was also reported in Western countries and Taiwan (2, 7, 9). Several features that apply to younger patients with T2DM may contribute to the poor glycemic control. Younger patients with T2DM are more likely to be busy with work and less motivated attend to their condition. Smoking, obesity, life style factors, lack of confidence in doctors, and poor compliance with medications are also more common among younger peoples (16). Consistent with the findings of reports elsewhere, the most common microorganism in patients with T2DM was KP (7, 14, 15), and a close association with PLA was observed in our present study. The pathophysiology of KP infection associated with DM is incompletely understood. However, the susceptibility of infection appears to be strongly associated with hyperglycemia Decreased mobilization of polymorphonuclear leukocytes, chemotaxis, and phagocytic activity may occur during hyperglycemia (17–19).

Possible sources reported for PLAs include biliary tract infections (such as cholangitis and cholecystitis), followed by portal venous disease, contiguous spread and hematogenous spread (20, 21). One study also showed that some infections almost always affect only patients with DM, such as gangrenous cholecystitis (22). Cholecystitis, the inflammation of the gallbladder is most commonly caused by the obstruction of the cystic duct by gallstone. Cholangitis is a bacterial infection that can occur when the common bile duct is blocked by gallstone. In our present study, we observed that the adjusted HR for PLAs in patients with T2DM was increased in individuals without gallstone, cholecystitis or cholangitis compared with in patients with PLA who did not have T2DM. T2DM is a strong risk for PLAs in patients without biliary tract disease.

Hepatic tuberculosis (TB) is uncommon and accounts for <1% of all tuberculous infection (23). Although there is an increased risk of tuberculosis and tuberculous abscess (tuberculoma) just in diabetic patients. It is difficult to diagnose hepatic TB in absence of previous history of TB or concurrent pulmonary involvement. Patients might be asymptomatic or present with non-specific constitutional symptoms such as fever, fatigue, weight loss, and night sweats (24). On cross-sectional imaging, hepatic TB can be comprehensively classified into micronodular and macronodular forms (25). Macronodules are more than 2 cm in size. Single macronodular hepatic TB is therefore at higher risk of being misdiagnosed as a neoplastic lesion or liver abscess (26). In this study, hepatic TB has been excluded based on occurrence of PLAs (ICD-9-CM 572.0) at inpatient record.

To our knowledge, this is the first study to examine where DM medication increases PLA risk in patients with T2DM. We considered all commonly used DM medications on the Taiwanese market during the study period, including metformin, glyburide, glimepiride, gliclazide, glipizide, glitazones, DPP4i, and acarbose. In our analysis, we found that 60.4% of patients were prescribed metformin, 36.5% were prescribed glimepiride, 31.2% were prescribed gliclazide, 23.7% were prescribed glyburide; acarbose, glitazones, glipizide, DPP4i and other medications were prescribed at rates of 16.9, 16.4, 15.8, 10.2, and 13.8% respectively. Metformin was the most frequently prescribed DM medication. Compared with comparison cohort, the patients with T2DM prescribing glyburide exhibited a significantly increased of PLA risk, whereas acarbose is potentially associated with a reduced PLA risk in patients with T2DM.

Our study provided evidence that compared with other DM medications, acarbose reduces the PLA risk in patients with T2DM. One large nationwide, population-based cohort study in Taiwan showed that, in patients with DM who used acarbose, colorectal cancer risk was reduced by 27% compared for patients with DM not using acarbose (27). Acarbose is an α-glucosidase inhibitors that delays the hydrolysis of disaccharides and polysaccharides to glucose in the small intestine. The mechanism for the prevention of colorectal cancer by acarbose is that acarbose increase more butyrate production by microbial fermentation after delayed carbohydrate absorption. Butyrate inhibits the growth of transformed cells and stimulates normal colonic mucosal proliferation (28). Another explanation for the reduction of colorectal cancer is that acarbose reduces colonic transit time and changes the fecal concentration of bile acids (29). A cohort study of 14,690 Taiwanese patients with PLA revealed an adjusted HR value for colorectal cancer of 5.5 for patients with PLA compared with controls (30). Therefore, acarbose may ostensibly reduce PLA risk in the newly diagnosed patients with T2DM.

Overall PLA incidence in the comparison cohort increase overtime, where the incidence in the DM cohort decreased from 4.02 in the first 2 years to 2.13 after 4 years of follow-up. The PLA occurrence in patient with DM was greatest at 0–2 years follow-up (HR = 4.02, 95% CI = 2.87–5.63). PLA always accompanied newly or recently diagnosed T2DM with poor glycemic control.

The current study was a large, representative, nationwide, population-based sample to explore PLA risk in patients with newly diagnosed T2DM. However, it has few limitations. First, our study group excluded the individuals younger than 18 years. Second, we could not identify other potential confounding factors in our database, such as smoking, alcohol consumption, nutritional status and environmental factors due to database limitation. Third, laboratory data such as blood glucose levels; hemoglobin A1c levels; liver function test results; imaging such as abdominal ultrasound or computed tomography; and blood cultures for KP, E. coli or other pathogens were unavailable. Other potential limitation to our study were selection bias or confounding by indication. Our selection bias and confounding by indication were patients with T1DM, age younger than 18 years old and other conditions such as poor immune conditions or medications for immune suppression.

In conclusion, PLA risk in patients with newly diagnosed T2DM was 2.83-fold higher than that in the comparison cohort. Younger patients and men with T2DM as well as patients with duration of DM <2 years had increased PLA risk. PLA risk was higher in newly diagnosed T2DM patients with gallstones and cholecystitis. In addition, compared with other DM medication, acarbose was associated with a lower PLA risk. Our findings highlight the value of active treatment strategies and close follow-up of patients with T2DM, and the effect of acarbose on PLA merits further investigation.

## Data Availability Statement

The datasets presented in this article are not readily available because the dataset used in this study is held by the Taiwan Ministry of Health and Welfare (MOHW). The Ministry of Health and Welfare must approve our application to access this data. Any researcher interested in accessing this dataset can submit an application form to the Ministry of Health and Welfare requesting access. Please contact the staff of MOHW (Email: stcarolwu@mohw.gov.tw) for further assistance. Taiwan Ministry of Health and Welfare Address: No.488, Sec. 6, Zhongxiao E. Rd., Nangang Dist., Taipei City 115, Taiwan (R.O.C.). Phone: +886-2-8590-6848. All relevant data are within the paper. Requests to access the datasets should be directed to Please contact the staff of MOHW (Email: stcarolwu@mohw.gov.tw) for further assistance.

## Ethics Statement

The studies involving human participants were reviewed and approved by the Ethics Review Board of China Medical University (CMUH104-REC2-115-AR4). Written informed consent for participation was not required for this study in accordance with the national legislation and the institutional requirements.

## Author Contributions

C-HK: administrative support. All authors have contributed significantly and are in agreement with the content of the manuscript, conception and design, collection and assembly of data, data analysis and interpretation, manuscript writing, and final approval of manuscript.

## Conflict of Interest

The authors declare that the research was conducted in the absence of any commercial or financial relationships that could be construed as a potential conflict of interest.
